# Scarf Osteotomy for Correction of Hallux Valgus Deformity in Adolescents

**DOI:** 10.1111/os.12539

**Published:** 2019-10-29

**Authors:** Xin‐wen Wang, Qian Wen, Yi Li, Cheng Liu, Kai Zhao, Hong‐mou Zhao, Xiao‐jun Liang

**Affiliations:** ^1^ Department of Foot and Ankle Surgery, Honghui Hospital Xi'an Jiaotong University Xi'an China; ^2^ Department of Prevention and Health Care Ninth Hospital of Xi'an Xi'an China

**Keywords:** Adolescent, Hallux Valgus, Osteotomy

## Abstract

**Objective:**

To report the radiological and clinical outcomes of the modified scarf osteotomy for the treatment of hallux valgus deformity in adolescents.

**Methods:**

This retrospective study analyzed 21 patients (31 feet) who underwent a modified scarf osteotomy for correcting juvenile hallux valgus deformity between March 2015 and January 2017. There were 3 male (3 feet) and 18 female (28 feet) patients. The average age at the time of surgery was 28.6 years (range, 20–35). Patients were postoperatively followed up in the outpatient department for 12–18 months. Clinical and radiological assessments were performed preoperatively and postoperatively at 1 year. Moreover, postoperative complications were recorded. Statistical analyses for differences between preoperative and postoperative values were performed.

**Results:**

All the 21 patients were postoperatively followed up for 12–18 months, with an average of 13.2 ± 2.5 months. Clinical assessment showed that the American Orthopaedic Foot and Ankle Society score was increased from preoperative 58.0 ± 5.8 to postoperative 94.2 ± 6.6 points, respectively, and the visual analog scale score was remarkably decreased from preoperative 6.0 ± 2.0 to postoperative 1.5 ± 2.0 points at 1 year follow‐up. Further radiological assessment showed that the hallux valgus angle was 37.5° ± 9.2°, 14.1° ± 6.5°, and 14.5° ± 6.5° before surgery, half a year after surgery, and 1 year after surgery, respectively; the intermetatarsal angle was 14.1° ± 4.4°, 4.8° ± 3.2°, and 5.5° ± 4.9°, respectively; and the distal metatarsal articular angle was 31.0° ± 3.5°, 7.2° ± 2.3°, and 7.5° ± 2.1°, respectively. They were significantly improved at half a year after surgery and 1 year after surgery compared to those before surgery. Complications occurred in two patients (9.5%) who had numbness on the skin of the edge of the medial incision, and the symptoms were relieved after 10 months. There was no clinical recurrence in all patients. One of the 31 feet had hallux varus, which was corrected in a second operation. Notably, a postoperative radiograph of a typical case whose both feet had hallux valgus deformity and underwent modified scarf osteotomy and additional Akin osteotomy showed adequate correction of the hallux valgus angle (HVA, 11°), intermetatarsal angle (IMA, 6°), and distal metatarsal articular angle (DMAA, 8°) on left foot compared to preoperative HVA (28°), IMA (13°), and DMAA (35°).

**Conclusion:**

The modified scarf osteotomy can effectively correct the adolescent hallux valgus deformity, which is worth popularizing.

## Introduction

Hallux valgus is a common deformity, leading to the formation of bunions and difficulty walking in footwear^1^. Hallux valgus deformity consists of medial deviation of the first metatarsal, and lateral deviation of the sesamoids^2^. The incidence of hallux valgus deformity is higher in China, probably due to genetic susceptibility, race, and ethnicity^3^. Hallux valgus deformity is reported to affect 22%–36% of adolescents, with a high recurrence rate up to 30%–40%^4^. Moreover, the incidence of juvenile hallux valgus is likely to increase if it is related to metatarsus adductus^5^. Although there are multiple osteotomies for correcting hallux valgus deformity in adults, the existence of open physeal growth plates limits the surgical correction of juvenile hallux valgus deformity^6^. The symptomatic juvenile hallux valgus deformity has become a challenge for the orthopaedic surgeon due to high recurrence rates^7^, ^8^. The high complication rates of operative treatment for adolescent hallux valgus deformity, such as recurrence and stiffness of the metatarsophalangeal joint, have gained genuine concern.

Compared with adult hallux valgus deformity, adolescent hallux valgus deformity is manifested as a remarkably increased distal metatarsal articular angle (DMAA) and the absence of medial subluxation^9^. Because the conservative management, such as shoe wear modifications, could not halt the progression of hallux valgus deformity^10^, the best treatment method is surgery and the choice of the surgery depends on the deformity degree. For severe deformities with an obvious abnormal DMAA, a diaphyseal osteotomy including scarf osteotomy that allows translation is considered as a better choice^11^, ^12^, ^13^. The scarf osteotomy is a Z‐step cut in the first metatarsal bone that can decrease the increased intermetatarsal angle (IMA) of hallux valgus deformity^14^. Since the description of scarf osteotomy by Burutaran^15^ in 1976, this procedure has been widely used for correction of hallux valgus deformities^16^, ^17^, ^18^. The scarf osteotomy is widely used in Europe because of its inherent stability and ease of internal fixation. This osteotomy allows horizontal displacement, shortening, rotation, elevation and lowering of the metatarsal head^19^. Compared to other shaft osteotomies, the advantages of this osteotomy are inherent stability and rigid compression at the osteotomy site, allowing immediate weight bearing and the option for bilaterality^19^. The scarf osteotomy also prevents shortening of the first ray and realizes early mobilization^20^. The indications for the scarf osteotomy range from mild to severe deformities, and the major indications include the increased IMA and DMAA, and symptomatic hallux valgus deformity^21^. A previous study has confirmed that scarf osteotomy has a better corrective ability for hallux valgus deformity compared with the distal metatarsal osteotomy^22^. Moreover, the scarf osteotomy has been suggested to treat adolescent hallux valgus deformity because this surgery allows rotational correction in addition to translation; however, mixed results were obtained from different studies^6^, ^23^, ^24^. In detail, John *et al*. demonstrated that scarf osteotomy was a safe and effective procedure for the treatment of juvenile and adolescent hallux valgus deformity^6^. Farrar *et al*. also indicated that the scarf osteotomy for adolescent hallux valgus achieved good outcomes, high levels of patient satisfaction, and a low level of symptomatic recurrence during early follow‐up^24^. Nevertheless, Zhou *et al*. suggested that the scarf osteotomy should be used with caution in the correction of symptomatic adolescent hallux valgus due to high recurrence rate. Furthermore, there is also uncertainty about the scarf osteotomy on the correction of DMAA^25^. The efficacy of scarf osteotomy on the correction of adolescent hallux valgus deformity merits further exploration.

In our clinical work, the scarf osteotomy was modified by carrying out wedge osteotomy in the distal end of osteotomy to reversely rotate the distal segment of the osteotomy. Moreover, the proximal phalanx (Akin) osteotomy has been widely used as an adjunct to the scarf osteotomy^26^, ^27^, ^28^, ^29^. After completing the scarf osteotomy and soft tissue reconstruction, the Akin osteotomy is always performed when the hallux is still more than 10° valgus^30^. The addition of Akin osteotomy can increase the corrective abilities of the scarf osteotomy^23^. An additional Akin osteotomy was also performed in our study if hallux valgus deformity was not completely corrected. Therefore, the purpose of the present retrospective study was as follows: (i) we aimed to report the clinical outcome of modified scarf osteotomy combined with or without Akin osteotomy in the treatment of hallux valgus deformity in adolescents; (ii) we tried to evaluate the radiological outcome of this modified scarf osteotomy in the treatment of adolescent hallux valgus deformity; and (iii) we intended to report the surgical skills or directions of this modified scarf osteotomy for correction of hallux valgus deformity in adolescents. Our findings will provide more evidence to support the clinical application of the modified scarf osteotomy in the management of adolescent hallux valgus.

## Materials and Methods

### *Patients*

Inclusion criteria for enrolling patients were as follows: (i) patients who were receiving modified scarf osteotomy for the first time as the primary hallux valgus surgery; (ii) patients who were diagnosed with severe hallux valgus: intermetatarsal angle (IMA) >11° or hallux valgus angle (HVA) >30° with an increased distal metatarsal articular angle (DMAA, ≥20°); (iii) patients who underwent a modified scarf osteotomy for correcting juvenile hallux valgus deformity between March 2015 and January 2017 in our institution; (iv) patients were postoperatively followed up in the outpatient department for 12–18 months; and (v) patients were retrospectively recruited.

Exclusion criteria were as follows: (i) patients with flat feet, rheumatoid arthritis, trauma history, tumor, mental illness, and pregnant or lactating women; (ii) patients with autoimmune diseases or blood disorders; (iii) patients who had incomplete follow‐up data; and (iv) patients with incomplete clinical data.

This retrospective study analyzed 21 patients (31 feet) who underwent a modified scarf osteotomy for correcting juvenile hallux valgus deformity between March 2015 and January 2017. Of these patients, 11 were bilateral. There were 3 male (3 feet) and 18 female (28 feet) patients. The average age at the time of surgery was 28.6 years (range, 20–35). The study was approved by the ethics review board of our hospital, and informed consent was obtained from all patients.

### *Surgical Technique*

**Figure 1:**
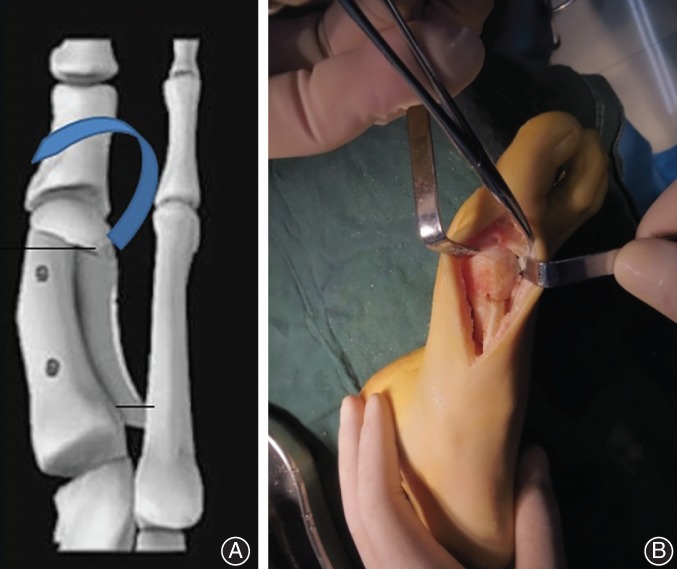
(A) Diagram of modified scarf osteotomy. (B) The distal articular surface of the metatarsal after reverse rotation.

1. Operations were performed under the condition of general anesthesia and peripheral nerve block (ankle block), and a pneumatic tourniquet at the root of the thighs was used. All patients were placed in the supine position.
2. The lateral soft‐tissues were completely released by a 2 cm dorsal incision between the first and second metatarsal heads. A 7 cm incision from the medial side of the first metatarsal was then made for removal of the exostosis inside the sagittal groove. Subsequently, “Z” osteotomy was then performed on the medial side of the first metatarsal. The lateral osteotomy line was located at the dorsal side of the metatarsal neck, and the metatarsal osteotomy surface was located at the metatarsal base. The proximal metatarsal was fixed with bone forceps and slightly pulled inward, making that the metatarsal head, and distal metatarsal were displaced outwards to correct IMA, with the maximum displacement reaching 2/3 of the diameter of the metatarsal shaft.
3. Wedge‐shaped osteotomy was performed at the distal end of the osteotomy, and then the distal bone block was rotated in reverse to straighten the metatarsal head and correct the DMAA that was the key point and difficulty in surgery (Fig. 1). After fixation with bone forceps, the bone block at the plantar side was inserted with two guide pins from the midline of the dorsal metatarsal. After confirmed that the angle was satisfactory under the X‐ray fluoroscopy machine, two 3.0 mm double‐threaded compression screws or absorbable screws were inserted for fixation, the distal protrusion of the first metatarsal generated by osteotomy was removed with a micro‐swing saw, and the osteophyte was trimmed and inserted into the osteotomy space.
4. The distal articular set angle (DASA) was then observed. If there was also hallux valgus deformity, an additional Akin osteotomy was performed. After confirming that internal fixation position was satisfied by X‐ray examination, it was decided whether the medial joint capsule was pulled according to the position of the metatarsophalangeal joint after osteotomy. If there was still a mild hallux valgus, the joint capsule would be pulled towards the proximal and dorsal sides, and the proximal bone of the osteotomy was sutured with absorbable suture. The incision was then rinsed and closed layer by layer.

### *Postoperative Care*

Postoperatively, the incision was pressurized and bandaged using an elastic bandage, and gauze strips were used between the first and second toes to isolate each toe after basic treatments, such as anti‐infection, detumescence, and analgesia. The affected foot was elevated, and each toe could be moderately active. Stitches were removed from the wound postoperatively at 2 weeks. The passive movement of the metatarsophalangeal joint was gradually strengthened 3 weeks after the surgery, and the X‐ray examination was performed 6 weeks after the surgery. If the osteotomy line had healed, the patients were allowed to walk in a shoe for weight bearing activities.

### *Clinical and Radiological Assessments*

Patients were postoperatively followed up in the outpatient department for 12–18 months. Clinical and radiological assessments were performed, and postoperative complications were recorded.

### *American Orthopedic Foot and Ankle Society (AOFAS) Scores*

Clinical evaluation was conducted preoperatively and postoperatively at 1 year. The clinical effects were evaluated with the scores on the hallux metatarsophalangeal interphalangeal scale developed by the American Orthopedic Foot and Ankle Society (AOFAS)^31^.

Forty points were assigned to pain, in which none was 40 points; mild or occasional was 30 points; moderate or daily was 20 points; and severe or almost always present was 0 point. Besides, 45 points were assigned to function, contained scales of activity limitations, footwear requirements, metatarsophalangeal (MTP) joint motion (dorsiflexion plus plantar flexion), interphalangeal (IP) joint motion (plantar flexion), MTP‐IP stability (all directions), and callus related to hallux MTP‐IP. In addition, 15 points were assigned to alignment (Good, hallux well aligned, 15 points; Fair or some degree of hallux malalignment observed or no symptoms, 8 points; Poor or obvious symptomatic malalignment, 0 points).

### *Visual Analog Scale (VAS)*

The results did not rely on imaging techniques. The perceived pain level was measured with a visual analog scale (VAS)^32^. The VAS pain scoring standard (scores from 0 to 10) was as following: 0 means painless; 1–3 means mild pain that the patient could endure; 4–6 means patient was in pain that could be endured and be able to sleep; and 7–10 means patient had intense pain and was unable to tolerate the pain. A score of 10 points is possible in a patient with no pain, full range of MTP and IP motion, no MTP or IP instability, good alignment, no limitation of daily or recreational activities, and no footwear limitations.

### *Radiographic Evaluation*

Radiographic evaluation was also conducted. Based on a standardized weight‐bearing anteroposterior radiograph of the foot, measurements of the IMA, HVA, and DMAA were performed during hospitalization and follow‐up.

The HVA was defined as the angle between the line from the center of the metatarsal base to the center of the first metatarsal head, and the line connecting the midpoints of the proximal and distal articular surfaces of the proximal phalanx.

The IMA was the angle between the line that connects the center of the base and head of the first metatarsal, and the line bisecting the diaphyseal portions of the second metatarsal^33^, ^34^.

The DMAA was the angle between the first metatarsal axis and the distal articular surface of the first metatarsal^35^.

### *Statistical Analysis*

Data conforming to a normal distribution were expressed as mean ± standard deviation (SD). The differences in the HVA, IMA, DMAA, VAS score, and AOFAS score between preoperative and postoperative checks were analyzed by a paired‐samples *t*‐test because these values were normally distributed. All statistical analyses were performed using SPSS (version 21.0; SPSS Inc., Chicago, IL, USA). A two‐sided probability value of *P* < 0.05 was considered statistically significant.

## Results

### *Baseline and Follow‐up*

In this study, 21 patients (31 feet) underwent a modified scarf osteotomy. All the affected feet had pain of bursitis, affecting walking, and there was no obvious osteoarthritis in the first metatarsophalangeal joint. Preoperative positive and lateral X‐ray images of weight‐bearing feet were taken and measured. All the 21 patients were postoperatively followed up for 12–18 months, with an average of 13.2 ± 2.5 months.

### *Clinical Evaluation Results*

#### *AOFAS*

Clinical assessment showed that the preoperative and 1 year postoperative AOFAS score was 58.0 ± 5.8 and 94.2 ± 6.6 points, respectively, and significant difference existed between them (*t* = 22.939, *P* < 0.001). The AOFAS score was remarkably improved after surgery, indicating an acceptable functional restoration of the forefoot postoperatively.

#### *VAS*

The VAS score was remarkably decreased from preoperative 6.0 ± 2.0 to postoperative 1.5 ± 2.0 points at 1 year follow‐up, indicating that the pain was significantly released postoperatively (*t* = 8.886, *P* < 0.001).

### *Radiological Assessment Results*

#### *HVA*

Radiological assessment showed that the HVA was 37.5° ± 9.2°, 14.1° ± 6.5°, and 14.5° ± 6.5° before surgery, half a year after surgery, and one year after surgery, respectively (*t* = 98.671, *P* < 0.001). They were significantly decreased half a year after surgery (23.4°) and 1 year after surgery (23.0°) compared to those before surgery.

#### *IMA*

The IMA was 14.1° ± 4.4°, 4.8° ± 3.2°, and 5.5° ± 4.9°, respectively (*t* = 46.532, *P* < 0.001). They were significantly decreased half a year after surgery (9.3°) and 1 year after surgery (8.6°) compared to those before surgery.

#### *DMAA*

The DMAA was 31.0° ± 3.5°, 7.2° ± 2.3°, and 7.5° ± 2.1°, respectively (*t* = 790.027, *P* < 0.001). They were significantly decreased half a year after surgery (23.8°) and 1 year after surgery (23.5°) compared to those before surgery.

### *Complications*

The wounds of patients healed well. Complications occurred in two patients (9.5%) who had numbness of the skin on the edge of the medial incision, and the symptoms were relieved after 10 months. There was no clinical recurrence in all patients. One of the 31 feet had hallux varus, which was corrected in a second operation. The reason was the medial articular capsule being sutured too tightly and the excessive pursuit of seed‐bone reduction.

### *A Case‐Report Result*

**Figure 2:**
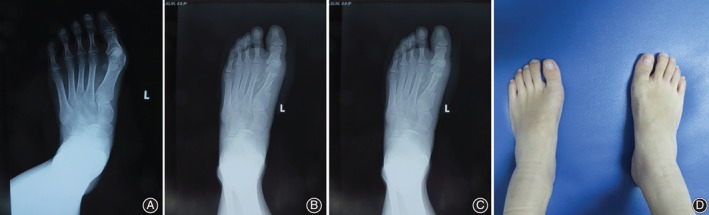
(A) A radiograph of preoperative weight‐bearing position shows hallux valgus deformity. (B) A radiograph of postoperative weight‐bearing position shows adequate correction of hallux valgus deformity. (C) A radiograph of weight‐bearing position 1 year after the surgery. (D) Outward observation of feet 1 year after the surgery.

Notably, a typical case (female, 23 years old) whose both feet had hallux valgus deformity was reported. The patient had pain in the hallux of both feet with limited shoe‐wearing activity for 3 years. The left foot of this patient underwent modified scarf osteotomy and additional Akin osteotomy. Absorbable screws were used for intraoperative fixation. A postoperative radiograph showed adequate correction of the HVA (11°), IMA (6°), and DMAA (8°) on left foot compared to preoperative HVA (28°), IMA (13°), and DMAA (35°) (Fig. 2). The patient's foot and toe functions recovered well, and the orthopaedic effect was satisfactory.

## Discussion

### *Summary of Major Results of Study and Comparison with Previous Studies*

In our study, the modified scarf osteotomy could effectively correct the DMAA, restore the matching of metatarsus and toe joints, and reduce the postoperative recurrence rate. Moreover, the addition of Akin osteotomy can increase the corrective abilities of the scarf osteotomy^23^. An additional Akin osteotomy was also performed in our study if hallux valgus deformity was not completely corrected. In this study, we took the frontal and lateral X‐ray in the standing position of the affected foot before the operation for measurement of the HVA, IMA, and DMAA. Subsequently, modified scarf osteotomy combined with or without Akin osteotomy was applied for the correction of hallux valgus deformity. The results showed that the modified scarf osteotomy had a significant improvement in the HVA, IMA, and DMAA of affected feet.

The AOFAS score was significantly increased postoperatively, and the VAS score was remarkably decreased. These data were consistent with previous findings that scarf osteotomy combined with Akin osteotomy is effective for hallux valgus in adolescents and postponement of correction until skeletal maturity is recommended due to a high recurrence rate following hallux valgus operation in children^4^. Taken together, we believe that the combination of the modified scarf combined with Akin osteotomies can effectively correct adolescent hallux valgus deformity.

### *Surgical Skills and Directions*

In the treatment of adolescent hallux valgus deformity with modified scarf osteotomy, several surgical skills or directions were obtained, as follows: (i) the osteotomy should be completed at one time with an oscillating saw to avoid difficulty in rotation caused by the unevenness of the osteotomy surface; (ii) in the longitudinal osteotomy, the tail of the micro‐swing saw was raised and the osteotomy was operated from the inner top to the outer bottom, otherwise the first metatarsal head may be raised, causing metastatic metatarsal pain^36^; (iii) the combination of scarf with Akin osteotomies could not affect blood supply and should be recommended; (iv) the medial cutaneous nerve should be protected due to the long medial incision, otherwise the incision will be painful for a long time after the operation. In this study, there were two patients with medial cutaneous nerve injury of the affected foot, resulting in numbness of the skin on the edge of the medial incision. The symptoms were relieved after 10 months; and (v) the main point of osteotomy was that the wedge‐shaped bone on the inner side of the distal end of the osteotomy was cut off, the proximal bone was pulled inward, and the distal end of the osteotomy was pushed outward to correct IMA. At the same time, the DMAA was corrected by reversely rotating the metatarsal head to match the first metatarsophalangeal joint. In this study, the average preoperative DMAA of 31.0° ± 3.5° remarkably improved to a postoperative angle of 7.5° ± 2.1°, indicating a better clinical outcome of the modified scarf osteotomy was obtained.

### *Limitations*

The small sample size, short follow‐up time, and the inherent deficiencies of retrospective study are the limitations of this study. More clinical practices with longer follow‐up data should be performed to confirm the efficiency of the modified scarf osteotomy.

### *Conclusions*

In summary, our data reveals that the modified scarf osteotomy combined with an Akin osteotomy can effectively correct the adolescent hallux valgus deformity. Because of better clinical efficacy and satisfaction, this osteotomy is worth popularizing for the treatment of hallux valgus deformity in adolescents.
